# Routine use of ancillary investigations in staging diffuse large B-cell lymphoma improves the International Prognostic Index (IPI)

**DOI:** 10.1186/1756-8722-2-49

**Published:** 2009-11-22

**Authors:** Dipti Talaulikar, Bruce Shadbolt, Jane E Dahlstrom, Anne McDonald

**Affiliations:** 1 Department of Haematology, The Canberra Hospital, Yamba Drive, Garran, Canberra, ACT, 2605, Australia; 2 Australian National University Medical School, Yamba Drive, Garran, Canberra, ACT, 2605, Australia; 3 Department of Epidemiology, The Canberra Hospital, Yamba Drive, Garran, Canberra, ACT, 2605, Australia; 4 Department of Anatomical Pathology, The Canberra Hospital, Yamba Drive, Garran, Canberra, ACT, 2605, Australia; 5 National Capital Private Hospital, Yamba Drive, Garran, Canberra, ACT, 2605, Australia

## Abstract

**Background:**

The International Prognostic Index (IPI) is used to determine prognosis in diffuse large B-cell lymphoma (DLBCL). One of the determinants of IPI is the stage of disease with bone marrow involvement being classified as stage IV. For the IPI, involvement on bone marrow is traditionally defined on the basis of histology with ancillary investigations used only in difficult cases to aid histological diagnosis. This study aimed to determine the effect of the routine use of flow cytometry, immunohistochemistry and molecular studies in bone marrow staging upon the IPI.

**Results:**

Bone marrow trephines of 156 histologically proven DLBCL cases at initial diagnosis were assessed on routine histology, and immunohistochemistry using two T-cell markers (CD45RO and CD3), two B-cell markers (CD20 and CD79a) and kappa and lambda light chains. Raw flow cytometry data on all samples were reanalysed and reinterpreted blindly. DNA extracted from archived paraffin-embedded trephine biopsy samples was used for immunoglobulin heavy chain and light chain gene rearrangement analysis. Using immunophenotyping (flow cytometry and immunohistochemistry), 30 (19.2%) cases were upstaged to stage IV. A further 8 (5.1%) cases were upstaged using molecular studies. A change in IPI was noted in 18 cases (11.5%) on immunophenotyping alone, and 22 (14.1%) cases on immunophenotyping and molecular testing. Comparison of two revised IPI models, 1) using immunophenotyping alone, and 2) using immunophenotyping with molecular studies, was performed with baseline IPI using a Cox regression model. It showed that the revised IPI model using immunophenotyping provides the best differentiation between the IPI categories.

**Conclusion:**

Improved bone marrow staging using flow cytometry and immunohistochemistry improves the predictive value of the IPI in patients with DLBCL and should be performed routinely in all cases.

## Background

Diffuse large B-cell lymphoma (DLBCL) is defined by the World Health Organization (WHO) as a heterogeneous entity, encompassing morphologic and genetic variants, and variable clinical presentations and outcomes [1]. It accounts for 80% of all aggressive lymphomas [1]. The median long-term overall survival in DLBCL is only ~40-50% [2] with variable outcomes depending on pre-treatment clinical and laboratory characteristics [3].

The International Prognostic Index (IPI) is a standard clinical tool that is widely used to predict outcome for patients with aggressive Non-Hodgkin lymphoma (NHL), including DLBCL. It uses a number of clinical and laboratory markers present at the time of diagnosis to predict survival. Age > 60 years, stage III/IV disease defined by results of radiological investigations and bone marrow (BM) biopsy, elevated lactate dehydrogenase (LDH) level, Eastern Cooperative Oncology Group (ECOG) performance status ≥ 2 and more than one extra nodal site of disease, are scored 1 each, and depending on the final score ranging from 0-5, 4 prognostic categories are created. These are: low risk correlating with IPI of 0-1, low-intermediate risk with IPI of 2, high-intermediate risk with IPI of 3, and high risk with IPI of 4-5. Five year overall survivals range from 73% to 26% [3]. However, limitations of the IPI are well recognised owing to the heterogeneity in clinical outcomes within IPI groups. Although gene expression profiling has been used to determine subtypes of DLBCL based on stages of B-cell differentiation [4], such studies are largely limited to the research setting. Efforts to improve clinical outcomes in DLBCL using reliable prognostic markers are ongoing [5,6]. In this study, we assessed the impact of improved staging investigations using easily available ancillary investigations on the IPI.

BM involvement was defined using histology alone in the large multicentre study from which the IPI was developed [3]. Ancillary tests such as flow cytometry, immunohistochemistry and molecular studies were not considered as part of staging towards the IPI. As these investigations have become more routinely available in laboratories around the world and their usage has increased, attempts have been made to define their clinical role. Currently the practice of performing ancillary tests is variable, and although several centres may perform at least some of these tests in routine practice, their usage is not appropriately validated and the impact of the routine use of these tests on the IPI has not been formally studied. When patients with histologically inapparent bone marrow involvement have positive results on ancillary tests, there is likely to be change in the IPI.

This study demonstrates that a significant change in the predictive value of the IPI can be brought about by incorporating ancillary investigations over and above routine histological diagnosis in staging bone marrows.

## Methods

### Patients

One hundred and fifty six retrospective cases diagnosed with histologically proven DLBCL at the Canberra Hospital from 1986-2005, on whom staging BM biopsies had been performed, were identified for the purpose of the study. After approval was obtained from the Australian Capital Territory (ACT) Human Research Ethics Committee, clinical information on patients was collected from the medical records department at The Canberra Hospital.

The average age of the patient cohort was 61 years (range 20-87 years), and the male to female ratio was 1.5:1. Baseline staging data using routine staging procedures [computed tomography (CT) scan, gallium/positron emission tomography (PET) scan and histological examination of BM] was available in 150 patients. Thirty nine (26%), 35 (23%), 45 (30%), and 31 (21%) were found to have stage I, stage II, stage III and stage IV disease respectively. Baseline assessment of IPI was possible in 148 patients. Thirty seven (25%) had an IPI of ≤ 1 of which 14 (9.5%) had an IPI of 0, and 23 (15.5%) an IPI of 1. IPIs of 2 and 3 were noted in 36 (24.3%) and 46 (31.1%) cases respectively. Twenty nine (19.6%) cases had an IPI of ≥ 4 of which 22 (14.9%) and 7 (4.7%) and an IPI of 4 and 5 respectively. The mean baseline IPI of the patient cohort was 2.41 with a standard deviation of 1.3.

Treatment data showed that of 152 patients on whom data was available, most were treated with anthracycline based regimens. One hundred and twenty nine patients (82.7%) were treated with Cyclophosphamide, Doxorubicin, Vincristine and Prednisolone (CHOP) [7] or variations of CHOP chemotherapy protocols [8,9]. Two patients were treated with Ifosphamide, Carboplatin and Etoposide (ICE) [10], 5 patients with Prednisolone, Etoposide and Novantrone (PEN) [11], 1 with Etoposide, Vincristine, Doxorubicin, Cyclophosphamide and Prednisolone (EPOCH) [12], 2 with Hyper-CVAD [13] comprising of hyperfractionated Cyclophosphamide, Vincristine, Doxorubicin and Prednisolone courses alternating with courses of Methotrexate and Cytarabine, 3 with Trans-Tasman Radiation Oncology group (TROG) protocol [14] and 1 with Methotrexate, Doxorubicin, Cyclophosphamide, Vincristine, Prednisolone and Bleomycin (MACOP-B) [15]. Nine patients were treated with palliative intent with steroids alone or in combination with non-anthracycline based drugs. Treatment details were not available in 2 patients and 2 were lost to follow-up. Thirty six patients (22.2%) received Rituximab. The median overall survival of the entire patient group was 6 years (95% confidence interval [CI]: 3.8, 8.4 years).

### BM histology

BM biopsies are performed as a routine assessment for all cases with DLBCL at first diagnosis in our institution. All trephines are fixed in buffered formalin and acetic acid for 24 hours and then decalcified using 5% nitric acid. Samples are then embedded in paraffin and sections stained with Haematoxylin and Eosin (H&E), giemsa stain and silver impregnation for reticulin. Archived H&E, giemsa and reticulin preparations on the trephine biopsy were retrieved for review. The mean trephine length for the patient cohort was similar to our previous reports, 17.6 mm with a range of 8-36 mm and the mean number of levels on H&E sections were 3.7 (range 1-8).

Two haematologists reviewed all slides blindly, and discrepant cases (n = 20) were resolved by consensus. Standardised criteria were used to classify trephine biopsy samples as positive, negative or indeterminate [16].

### Flow cytometry

Raw immunophenotypic data on all bone marrow biopsies was retrieved from laboratory records and re-analysed.

Multiparametric flow cytometric analysis is performed in our laboratory, with marrow cells immunophenotypically labelled by direct four-colour immunofluorescence using a panel of antibodies (CD45, CD19, CD20, CD22, CD10, HLA-DR, Kappa, Lambda, CD2, CD3, CD5, CD7; Becton-Dickinson).

Red cells are lysed by incubation with ammonium chloride, and cells washed in phosphate buffered saline after centrifugation. A cell-suspension of 1 × 10^6 ^cells per tube is incubated with the monoclonal antibody at room temperature, then washed and resuspended in a solution of phosphate buffered saline and foetal calf serum. Isotypic controls used are IgG1 and IgG2.

Data acquisition is on a Becton-Dickinson flow cytometer with a minimum of 2000 lymphocytes counted in each sample. Bright CD45 fluorescent staining and intermediate side scatter are employed as the primary gating strategies to identify the lymphocyte population, and further gating is performed as required based on cell size or using back gating on CD19 positive events.

Previously archived raw data were reanalysed, including blinded re-determination of the population of lymphocytes to be gated. Positive results on flow cytometry were defined as light chain clonal restriction with a kappa: lambda ratio of >3:1 or <0.3:1[17]. Predominance of B-cells in the gated population alone without light chain restriction was not considered as a positive result.

### Immunohistochemistry

Immunohistochemical analysis was performed on a Ventana Benchmark NexES machine. Sections from archived formalin-fixed decalcified paraffin-embedded (FFDPE) trephine biopsies were immunostained using the streptovidin-biotin method. The following monoclonal antibodies were used: CD3 [Dako clone CD3, 1:200 dilution], CD45RO [Novacastra clone UCLH-1, 1:1000 dilution], CD20 [Zymed clone L26, 1:50 dilution], CD79a [Dako clone JCB117, 1:500 dilution], Kappa [Novacastra clone kp-53, 1:750 dilution], and Lambda [Novacastra clone Hp-6054, 1:750 dilution]. All antibodies are validated and routinely used in our laboratory. CD20 and CD3 are reported to be sensitive at assigning lineage in diffuse aggressive NHL [18] and CD79a and CD45RO were selected over others owing to familiarity and to maintain consistency. These are the antibodies used for diagnostic tissue sections in our laboratory. Heat retrieval was used for all antibodies and tonsillar tissue was used as a positive control. A standardised system of reporting was adopted and was followed for all stains by two pathologists blinded to previous assessment on histology.

Features used to define involvement on immunohistochemistry reflected standardised histology criteria. The presence of B-cell aggregates was classified as abnormal or malignant when there were large numbers of aggregates, the aggregates were large-sized, or contained disproportionate numbers of larger cells. Controls (six morphologically normal marrows) were used to create a visual impression of normal amounts of background T and B-cells. Scattered small or large B-cells were classified positive only when the numbers were substantially increased as compared to controls. A conservative approach was adopted to avoid false positives. Discrepancies between the two pathologists were resolved consensually.

### Molecular studies

Samples for molecular studies were obtained from formalin-fixed decalcified paraffin-embedded (FFDPE) trephine sections. DNA extraction was performed manually using the Roche High Pure PCR Template Preparation Kit from two 10-micron FFDPE trephine sections according to the manufacturer's instructions. To verify the integrity of the DNA extracted from the paraffin sections, and to validate results, all samples were amplified with the control master mix provided in the Immunoglobulin heavy chain (IgH) gene clonality kit from Invivo Scribe Technologies based on the BIOMED2 protocols (IgH Gene Clonality Assay - Gel Detection; InVivo Scribe Teachnologies, USA). This is a multiplex PCR that targets multiple genes and generates a series of amplicons 100, 200, 300, 400 and 600 base pairs (bp) in length.

IgH gene rearrangement analysis was performed on all cases, targeting the conserved framework regions (FR) FR1 [IGH_A_: V_H_FR1-J_H_] and FR3 [IGH_C_: V_H_FR3-J_H_] using the Invivo scribe kit based on the BIOMED2 protocols [19]. Only FR1 and FR3 were analysed owing to limited amounts of DNA and based on reports from other groups [20]. This was combined with light chain gene rearrangement analysis and included two reactions targeting Ig Kappa (IgK) variable and joining regions [IGHK_A_: V_k_-J_k_;] and IgK variable and intragenic regions [IGK_B_: V_k_-K_de_]. The PCR reactions consisted of 45 μL of the FR1, FR3 or IGK master mix solution, 2.5 units of Amplitaq Gold (Applied Biosystems, USA) and 5 μl of template DNA (with an average template DNA concentration of 300-400 ng/μl). Thermo cycling was performed according to the kit protocol with no modifications on a Perkin Elmer 9600 thermocycler. Controls consisted of a positive DNA control, negative extraction control and negative PCR control. Water was used as a negative control in both cases.

Non-denaturing polyacrylamide gel electrophoresis was used to resolve the FR1 and FR3 PCR products. 25 uL of PCR product was loaded onto a 6% polyacrylamide gel and 250 V applied for 1.25 hours for FR1 and 1.5 hours for FR3 reactions. After electrophoresis, the gels were stained with ethidium bromide and visualised under UV light.

For the IGK reactions, PCR products were denatured at 94°C for five minutes and subsequently cooled at 4°C for 60 minutes to induce duplex formation. Non-denaturing polyacrylamide gel electrophoresis was used to resolve the PCR products. 25 uL of PCR product was loaded onto a 6% polyacrylamide gel and 250 V applied for 1.5 hours each for both reactions.

FR1, FR3 and IgK gene rearrangements were reported as clonal, polyclonal or not detected. The expected sizes of the PCR products were 310-360 bp for FR1 and 100-170 bp for FR3 which together are estimated to account for approximately 70% of all rearrangements [20] IGK PCR products were expected to be in the following ranges: 120-160 bp, 190-210 bp, 260-300 bp for IgKA and 210-250, 270-300, 350-390 bp for IgKB.

### Statistical analysis

Survival data were recorded for each patient. Besides descriptive analysis, Kaplan Meier curves were created with cumulative survival as the outcome. Forward stepwise multivariate Cox regression analysis using the likelihood ratio method was used to establish a comparison between baseline IPI and two revised IPI models. The first model (rIPI1) was based on routine use of immunophenotyping alone (flow cytometry and immunohistochemistry) and the second (rIPI2) was based on routine use of immunophenotyping and molecular results. A probability of 0.05 was used as the entry criterion and 0.1 was considered for removal. Patients treated with palliative intent were excluded from all survival analyses. All analyses were performed using the software programme Statistical Package for Social Sciences (SPSS) version 14.0.

## Results

### Histology

Of the 156 cases on which bone marrow histology slides were available, 24 were positive on routine histology. Six cases were reported as indeterminate using Cheson criteria, and agreed upon as being positive for involvement after consensual review. H&E stains showed no evidence of involvement in 126 cases.

### Immunophenotyping

Flow cytometry data was evaluable in 152 cases, of which 27 (17.3%) cases were noted to be positive for involvement using standardised light chain ratios. Ten of these 27 cases were also positive on routine histology.

Immunohistochemistry using T and B-cell markers showed involvement in 43 cases of 154 available cases of which involvement on routine histology was noted in 25/42 cases. One case was not comparable due to loss of H&E slides. Flow cytometry and immunohistochemistry each detected histologically inapparent involvement in 17 cases (11%).

### Molecular studies

Amplification was obtained in 133/155 cases (84.7%) with amplification at 96 base pairs (BP), 200 bp, 300 bp, 400 bp and 600 bp noted in 125 (79.6%), 74 (47.1%), 32 (20.4%), 25 (15.9%) and 18 (11.5%) cases respectively. Forty one cases of 155 evaluable ones were positive on immunoglobulin heavy and light chain gene analysis. Three showed no amplification on amplification controls. Thirty four cases were positive on light chain analysis with all showing a clonal band with kappa A; three cases also showed clonal reactions with kappa B. Overall, 19 cases were positive on heavy chain analysis (FR3: 18 cases, and FR1: 4 cases) Of these, three cases were positive on both reactions. Overlap with light chain analysis is shown in table 1. Overall, 12/41 cases were positive and 29 negative on routine histology.

**Table 1:** Results on immunoglobulin heavy chain (IgH) and light chain (IgL) gene rearrangement studies

	#IGK +ve	IgK -ve	Total
*FR3 +ve	13	5	**18**

**FR3 -ve	21	116	**137**

**Total**	**34**	**121**	**155**

FR1 +ve	2	2	**4**

FR1 -ve	32	119	**151**

**Total**	**34**	**121**	**155**

* FR1: framework I

** FR3: framework III

# IGK: immunoglobulin kappa

To establish tumour origin, DNA was extracted from 17 available primary FFDPE tissue blocks and gene rearrangement analysis performed. Comparable clonal bands could be identified in only 10 cases. Of these, 2 were positive on routine histology.

Thus, using stringent criteria to account for false positivity, routine molecular staging on FFDPE trephine biopsy tissue yielded positive results in eight (5.1%) histologically negative cases.

### Effect on stage and IPI

Thirty cases were upstaged using immunophenotyping alone with 6 cases upstaged from stage I to IV, 12 from stage II to IV, and 12 from stage III to IV. When molecular results were added, two additional cases were upstaged from stage I, 2 from stage II and 4 from stage III.

Two new revised IPI (rIPI) models were computed for all cases. The first (rIPI1) was based on immunophenotyping results alone i.e. flow cytometry and immunohistochemistry and the second (rIPI2) on immunophenotyping and molecular results. Changes to the IPI essentially occurred when stage of disease was upgraded from I or II to stage IV diseases. Of 148 cases where IPI and rIPI were assessable, three cases were upgraded from IPI 0 to a rIPI1 of 1, 4 cases of IPI 1 changed to a rIPI of 2, 5 cases of IPI 2 were upgraded and 6 cases of IPI 3. No changes were noted in IPI 4-5 group. Overall, 18 patients had a change in their IPI. Of these, three changed their IPI from 0 to 1, which was not apparent when only four prognostic categories were considered. Incorporating molecular results, rIPI2 was found to be upgraded in 6, 7 and 6 cases of IPI 0-1, 2 and 3 respectively.

### Survival

**Figure 1:**
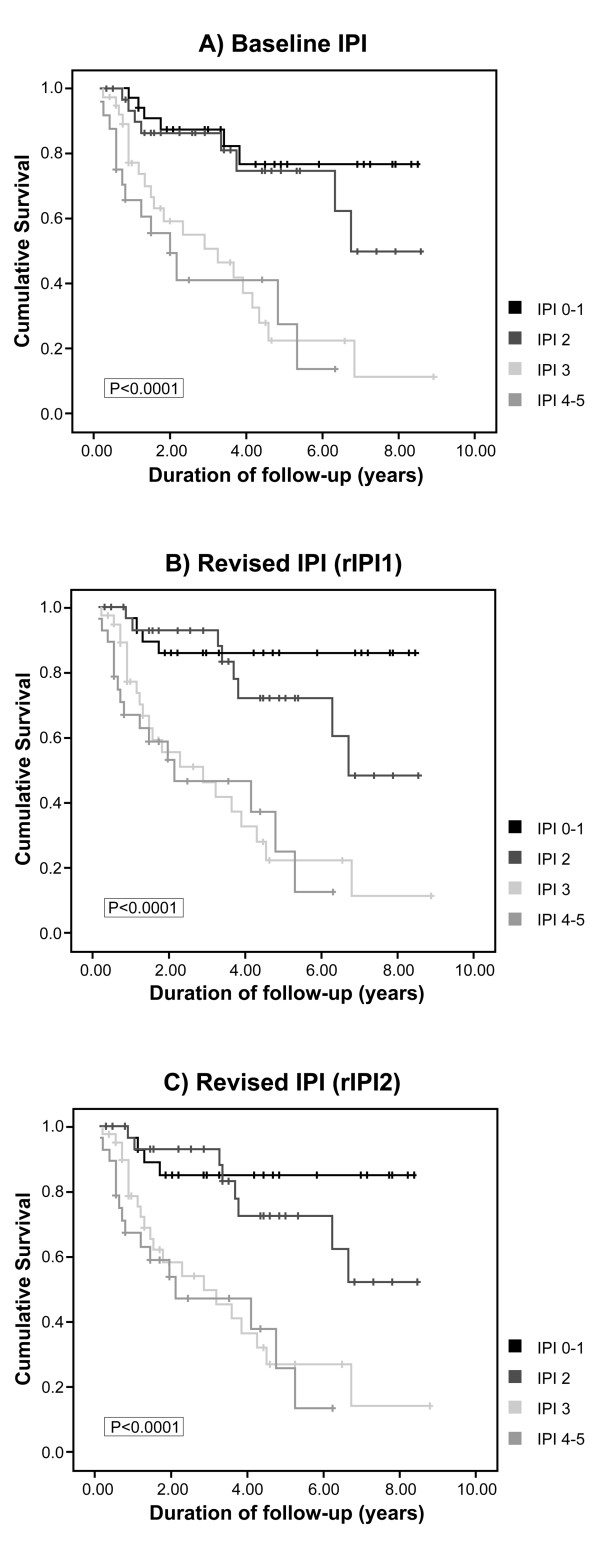
**The three Kaplan Meier curves show differences in cumulative survival between with low-risk, low-intermediate, high-intermediate and high-risk categories using a baseline IPI model (A) and two revised IPI models**. The first (rIPI1) incorporates flow cytometry and immunohistochemistry as routine staging **(B) **and the second IPI model (rIPI2) additionally incorporates molecular testing using IgH/IgL analysis **(C)**.

Kaplan Meier curves were created to assess the impact of baseline IPI and the two new revised IPI models rIPI1 and rIPI2 on overall survival. Figure 1 shows the cumulative survival of the four IPI categories using a baseline IPI model, a revised model using immunophenotyping (rIPI1) and a revised model incorporating molecular studies and immunophenotyping (rIPI2) respectively. All three models were statistically significant with p values of < 0.0001.

### Multivariate analysis

Using a multivariate forward stepwise (likelihood ratio method) Cox regression, the three IPI models were competitively considered for their contribution to predicting survival. The score tests before inclusion into the model were: Baseline IPI: 28.5 (df = 3, P < 0.001), rIPI1: 31.2 (df = 3, p < 0.001) and rIPI2: 27.9 (df = 3, p < 0.001). The revised rIPI1 model was then entered into the regression model. The hazard ratios of dying relative to the 0/1 IPI prognostic categories were: rIPI1 category 2 = 2.0 [95% CI, 0.61, 6.76, p = 0.248], rIPI1 category 3 = 7.6 [95% CI, 2.61, 22.36, p < 0.0001], rIPI1 category 4/5 = 8.9 [95% CI, 2.94, 27.17, p < 0.0001]. The baseline IPI model and rIPI2 models were excluded from the regression model because they did not further contribute to explaining survival [p = 0.5, p = 0.6 respectively].

Table 2 provides a summary of the relative performances of the three IPI models in predicting survival. It can be seen that rIPI1 provides the best differentiation between the IPI categories and the largest point estimate hazard ratios.

**Table 2:** Summary table showing the hazard ratios from Cox regression analyses for the three IPI models, baseline IPI, rIPI1 and rIPI2

	**rIPI1**^#^ Hazard ratios (95% CI)	**Baseline IPI**^**†**^ Hazard ratios (95% CI)	**rIPI2**^**‡**^ Hazard ratios (95% CI)
Low-risk IPI (score 0/1)	Reference	Reference	Reference

Low-intermediate IPI (score 2)	2.0 (0.61, 6.76) p = 0.248	1.56 (0.54, 4.51) P = 0.4	1.8 (0.5, 6.0) P = 0.3

High-intermediate IPI (score 3)	7.6 (2.61, 22.36) p < 0.0001	5.32 (2.14, 13.22) P < 0.0001	6.4 (2.2, 18.6) P = 0.001

High-risk IPI (score 4/5)	8.9 (2.94, 27.17) p < 0.0001	6.9 (2.61, 18.32) P < 0.0001	8.1 (2.7, 24.6) P < 0.001

^#^Cox regression using forward likelihood ratio method (X^2^: 31.5, df = 3, p < 0.0001)

^†^Cox regression entering baseline IPI into the model first (X^2^: 27.4, df = 3, p < 0.0001)

^‡^Cox regression entering rIPI2 in to the model first (X^2^: 28.2, df = 3, p < 0.0001)

To study the effect of treatment with Rituximab on survival, this too was considered, but not found to contribute significantly to the Cox regression model (p:0.96).

## Discussion

In this study, we have shown a significant improvement in the predictive value of the IPI using ancillary staging investigations, particularly immunophenotyping, on the BM. By upstaging a proportion of cases, routine use of immunophenotyping provides better differentiation across the IPI prognostic categories. This has been confirmed using clinical outcomes in this study. These results validate the current guidelines that recommend incorporating immunophenotyping in routine staging and suggest the use of a new and more inclusive definition of BM involvement within the IPI.

There have been several previous studies on the clinical role of ancillary investigations such as flow cytometry [17,21-25], and IHC [17,26,27] in NHL, although variable results are noted depending on the histological subtypes of NHL.

Overall, multiparametric flow cytometry has been reported to be more sensitive than histology alone, and detection of flow cytometry positive cases have been reported in 3-11% of histologically negative cases, with rates in DLBCL varying from negligible to ~15% [17,21-23,25]. The converse is also true and 5-20% histologically positive DLBCL cases have been reported to be negative on flow cytometry [17,21,22,25]. This may relate to a number of factors such as sampling and adequacy of histological diagnosis. Further development of multicolour flow cytometry (6, 8 or 10 colour flow cytometry) and its introduction into the clinical laboratory is likely to further improve the sensitivity of this technique.

Similarly, IHC is reported to detect marrow involvement in histologically negative cases in ~10-23% of cases depending on the histological diagnosis and the antibodies used [26,27]. This is considered to be due to examination of a greater number of levels and also to easier detection of scattered malignant cells within normal haemopoietic tissue.

Overall, we found that use of immunophenotyping i.e. flow cytometry and immunohistochemistry in staging bone marrow biopsies upstages ~20-22% of patients with DLBCL. The two investigations complement each other. Flow cytometry is generally performed on aspirate samples and can be expected to add independent prognostic value. Immunohistochemistry, on the other hand, is performed on the trephine. Although it does not add independent prognostic value, it is a more sensitive technique than histology alone. This study shows the value of incorporating these tests as a routine rather than using them in histologically ambiguous cases only. Better differentiation into low-risk, low-intermediate, high-intermediate and high-risk IPI categories is obtained by using these tests on all staging bone marrows.

There are several previous studies addressing the role of gene rearrangement (IgH/IgL) studies [28-31] in NHL. Of particular interest is the study by Mitterbauer-Hollander et al which showed 16% of histologically negative cases had clonal IgH and/or IgL genes within the bone marrow [31]. The authors demonstrated a significant difference in overall survival at 5 years amongst patients with positive histology and molecular studies, negative histology but positive molecular studies, and negative histology and molecular studies. In our study, only ~5% of histologically negative cases were found to have rearranged immunoglobulin genes. We were unable to demonstrate a difference in overall survival or a change in the predictive value of the IPI by inclusion of molecular staging. This is likely to be related to the unavailability of archived fresh frozen trephine tissue or DNA for our study resulting in all molecular analyses being performed on FFDPE trephine tissue. This is in contrast to the previous study, in which all molecular analyses were performed on fresh bone marrow aspirates [31]. It is well known that fresh tissue yields better quality DNA compared to FFDPE tissue [32]. It should also be noted that the BIOMED2 based protocols are not as well established on FFDPE tissue [19], although occasional groups have modified the protocols with improved results [33]. It may be of interest to determine if the use of such modified protocols would improve the prognostic significance of molecular staging on FFDPE tissue. Other alternatives to PCR staging may be staging using Fluorescent in-situ hybridisation (FISH) probes with some recent literature demonstrating that FISH using IgH/BCL2 may give improved results as compared to PCR on paraffin-embedded sections [34].

Besides the obvious advantage of availability of archived trephine biopsy tissue, the other reason for choosing to perform molecular staging on trephine biopsy rather than aspirate is that histological bone marrow involvement is noted more commonly on trephine biopsies. This has been demonstrated in previous studies [35] and largely attributed to sampling and the tendency of lymphoma cells to adhere to bony trabeculae [36]. As such, there would be greater likelihood of detecting clonal gene rearrangements on trephine biopsy rather than aspirate samples. Collecting additional trephine biopsy samples for such testing may be logistically difficult. Improved DNA extraction methods and optimal modification of the BIOMED2 protocols for FFDPE trephine tissue may be the best realistic option.

There are several limitations of our study. This is a small retrospective study in DLBCL cases at initial diagnosis. As only a proportion of cases were treated with Rituximab in this study, we used multivariate analysis to demonstrate that it was not a significant confounding factor in our analysis. However, we acknowledge a prospective study may be required to confirm that the results are valid in Rituximab treated patients. The other major limitation is the use of archived rather than fresh trephine tissue for molecular staging due to the logistics of obtaining fresh trephine tissue in our centre. We acknowledge that we may have been able to demonstrate improvement in the prognostic significance of the IPI using gene rearrangement studies if fresh tissue had been analysed.

Despite the limitations of our study, we were able to demonstrate an improvement in the prognostic significance of the IPI by use of simple, relatively inexpensive and readily available staging investigations such as flow cytometry and IHC. Our results suggest that a large prospective study is warranted to assess the impact of staging investigations on the IPI in a more homogenously treated DLBCL population.

## Conclusion

• The predictive value of the IPI can be improved significantly by the routine use of immunophenotyping on staging bone marrow biopsy.

• Immunophenotyping i.e. flow cytometry and immunohistochemistry should be recommended as routine investigations on all bone marrows at initial diagnosis, as the detection of occult disease in morphologically normal marrow affects clinical outcome in DLBCL.

• In this study, molecular analysis did not further contribute in improving the prognostic significance of the IPI. This is likely to have been due to technical limitations.

• Larger prospective studies are warranted to assess the impact of staging investigations including gene rearrangement studies on the IPI in a more homogenously treated DLBCL population.

## List of abbreviations

BM: bone marrow; CT: computed tomography; DLBCL: diffuse large B-cell lymphoma; ECOG: eastern cooperative oncology group; FISH: fluorescent in-situ hybridisation; FR: framework; IG: immunoglobulin; IPI: International prognostic index; LDH: lactate dehydrogenase; NHL: Non Hodgkin Lymphoma; PET: positron emission tomography; rIPI: revised International prognostic index; WHO: World Health Organization.

## Conflict of interests

The authors declare that they have no competing interests.

## Authors' contributions

DT: project design, reporting histology, flow cytometry and immunohistochemistry. Performing all molecular analyses and interpreting results. Data entry and basic statistical analysis. Writing the paper. BS: input into project design, help set up database, advice on basic statistical analysis and performance of survival analyses. Input into and final approval of paper. JD: input into project design, designing standardised reporting format for immunohistochemistry, and blinded review of immunohistochemistry. Input into and final approval of paper. AM: reporting of histology slides, input into and approval of final paper.
